# Enhancing the health and well-being of international students: insights from changes in their lifestyle post-COVID-19

**DOI:** 10.3389/fpubh.2024.1470378

**Published:** 2024-12-17

**Authors:** Jingru Ma, Kazuya Saita, Fumiko Kaneko, Hitoshi Okamura

**Affiliations:** Graduate School of Biomedical and Health Sciences, Hiroshima University, Hiroshima, Japan

**Keywords:** international student, COVID-19, post-pandemic, public health, well-being, immigrants

## Abstract

**Introduction:**

The COVID-19 pandemic’s global impact has been profound, particularly for vulnerable populations, such as asylum seekers, refugees, and immigrants. Likewise, international students, who fall under the immigrant category umbrella, have faced considerable challenges throughout the pandemic. This study aimed to identify insights for enhancing Japanese international students’ health and well-being by investigating how epidemic prevention policies implemented by schools and the government influenced changes in their lifestyles during the post-pandemic era.

**Methods:**

Semi-structured interviews were conducted and inductive thematic analysis performed using NVivo software, to investigate the lifestyle changes of 20 (8 male and 12 female) international students (mean age: 31.5 years) at Hiroshima University, Japan, during the post-pandemic era.

**Results:**

The analysis revealed alterations in lifestyle habits and interpersonal relationships in the aftermath of the pandemic, with most international students continuing to practice some of the epidemic prevention and health behaviors they had adopted during the pandemic, including handwashing (*n* = 10), mask-wearing (*n* = 12), and physical distancing (*n* = 4), as a matter of habit. However, some of these epidemic prevention and health behaviors have not persisted as habits after the pandemic. However, there has been an increased emphasis on maintaining family relationships (*n* = 5).

**Discussion:**

Addressing language and information dissemination barriers that international students may encounter when accessing medical services, and providing them with employment guidance and support more suited to their current situation, are conducive to enhancing their health and well-being. Additionally, enhancing international students’ public awareness is crucial for safeguarding their health and preparing them for potential future public health emergencies.

## 1 Introduction

Certain immigrants within host countries face marginalization stemming from social conditions (1). Despite the World Health Organization’s advocacy for universal health coverage, aimed at guaranteeing unfettered access to superior healthcare services devoid of financial constraints, immigrants frequently find themselves marginalized by host nations owing to barriers like language and cultural disparities (2). The emergence of the novel coronavirus (COVID-19 or 2019-nCoV or SARS-CoV-2) in December 2019, marked the onset of a multifaceted and continuously evolving global health crisis, profoundly affecting all facets of society (3). Globally, the COVID-19 pandemic’s ramifications have been disproportionately borne by asylum seekers, refugees, and migrants (4). Improving immigrants’ health and well-being is crucial for enhancing their quality of life and happiness, as well as for controlling pandemics.

In Japan, the government implemented non-coercive (mild) lockdown measures. In response to the rapid spread of the virus, the Japanese government declared a month-long state of emergency on April 7, 2020, urging residents to limit outdoor activities only to those essential, for maintaining their daily lives, while adhering to infection prevention measures (5). Unlike many other countries, Japan’s emergency declaration was not legally binding on the public. The Japanese government, in its response to the COVID-19 pandemic and other disasters, encourages individuals to collectively assume responsibility, and undertake all possible measures to mitigate their impact (6). This involves controlling the epidemic through voluntary self-restraint, and spontaneously adopting preventive behaviors (7). Thus, individual preventive behaviors significantly influence epidemic and disaster control. The preventive behaviors undertaken by Japanese immigrants are pivotal in addressing both epidemics and disasters. As a mono-ethnic country, Japan lacks diversity; therefore, it is crucial to improve the health and well-being of immigrants residing in Japan.

The Japan Immigration Service Agency’s statistics indicated that as of the end of June 2022 international students constituted 8.8% of the immigrant population (8). The Japanese Ministry of Education has consistently implemented a program aimed at promoting international students’ enrollment (9). Moreover, there has been a steady annual increase in the number of international students in Japan (10). Studying abroad, as a form of immigration, should also not be disregarded. Research on the health and well-being of foreign immigrants during the COVID-19 pandemic has been conducted (11–13). However, studies in Japan, focusing on the health and well-being of foreign immigrants, particularly international students, in the post-pandemic period, remain limited.

This study’s purpose was to conduct an in-depth analysis of the lifestyle changes among international students in the post-pandemic period, influenced by epidemic prevention policies implemented by both schools and the government, and also to identify insights that could enhance their health and well-being. It aimed to offer essential references and foundations for developing targeted and effective health promotion measures.

## 2 Materials and methods

This study aimed to explore the lifestyle changes experienced by international students studying in Japan during the post-pandemic period, particularly in light of the epidemic prevention policies implemented by educational institutions and the government. By employing a qualitative research design, this study sought to capture the nuanced perspectives of international students regarding the impact of these policies on their daily lives. The use of semi-structured interviews allows for an in-depth exploration of participants’ experiences, facilitating a rich dialogue that can reveal new insights into their adaptations and challenges.

### 2.1 Questionnaire development

This qualitative study employed semi-structured interviews to facilitate an in-depth exploration of participants’ experiences. The research team convened a meeting to discuss and develop a questionnaire comprising 6 questions. This questionnaire enabled the researcher to ensure more systematic interview procedures for participants while concurrently exploring new areas of dialogue. One participant underwent a pilot test of the questionnaire, and after incorporating their feedback, the questionnaire was modified. Subsequently, the participant was invited for a second interview.

### 2.2 Participants

To investigate shifts in lifestyles among international students post COVID-19, influenced by epidemic prevention policies from schools and the government, the sample size was determined based on previous studies. Prior research suggests that sample sizes ranging from 6 to 8 individuals are optimal for maintaining sample homogeneity, while sample sizes between 12 and 20 individuals may better accommodate heterogeneity (14). This study focused on a cohort of international students in Japan, deliberately avoiding constraints imposed by specific demographic traits, such as marital status, gender, or age, thus leveraging the richness of diverse data. Consequently, 20 international students were recruited from Hiroshima University. As the first author was an international student at Hiroshima University, the research invitation letter was sent to the Hiroshima University international student chat group with the assistance of a Bangladeshi student who knew the first author, and a Chinese student who was also acquainted with the first author. Participants interested in the study were encouraged to contact the first author either by phone or email, using the contact information provided in the invitation letter. The snowball sampling method was employed with the expectation that participants who had accepted the invitation could refer additional eligible participants.

### 2.3 Interviews

From April to June 2023, 19 participants took part in face-to-face semi-structured individual interviews at Hiroshima University. One participant who had given birth during the study period, requested a phone interview from home. The first author took field notes during these interviews. To ensure participant privacy, only the participants and researchers (no non-participants) were present during the interviews.

All interviews, conducted by the first author were audio-recorded with the participants’ consent. Prior to commencing the interview, participants received a concise introduction outlining the research’s purpose, significance, methods, and relevant content. The first author had previously undertaken courses related to qualitative research, and this study was conducted with the guidance of a doctoral supervisor, possessing extensive experience in qualitative research and qualifications in psychiatry, to ensure its credibility and validity. Chinese participants were interviewed in their native language, while others were interviewed in English. Given the English proficiency requirement for admission to Hiroshima University, all participants could communicate in English to varying extents. The first author, with a background in translation studies and fluency in Chinese, Japanese, and English, encountered no significant language barriers during the interviews. Nonetheless, to foster a relaxed atmosphere and elicit more intuitive responses from Chinese participants, the interviews were conducted in Chinese.

The first author conducted interviews with participants using a questionnaire comprising six questions. The interview questions were as follows: (1) Are you aware of the prevention policies implemented by schools and the Japanese government to ensure your safety and well-being during the pandemic? (2) Can you describe the specific COVID-19 prevention policies implemented by schools and the Japanese government? (3) How do you obtain information on pandemic prevention and health? (4) Did you voluntarily follow these preventive measures during the pandemic? (5) Under the influence of epidemic prevention policies implemented by the Japanese government and schools, has your lifestyle changed after the end of COVID-19? (6) What specific changes have occurred in your lifestyle?

The interviews lasted from 0.5 h to 1 h, with the length varying according to personal circumstances and experiences. All interviews adhered to the Consolidated Criteria for Reporting Qualitative Research Checklist (15). Additionally, Guba’s four criteria—credibility, transferability, dependability, and confirmability—were used to ensure trustworthiness (16).

### 2.4 Data analysis

Interview recordings were converted into text using Microsoft Word’s speech-to-text feature. The transcribed text was then scrutinized and refined to rectify any inaccuracies in software recognition. Subsequently, the first author translated the Chinese interviews into English. Following this translation, the research team, composed of two males and two females, proceeded with the data analysis. The research team included a doctoral supervisor with extensive experience in qualitative research and qualifications in psychiatry, a university lecturer specializing in social welfare studies, a university assistant qualified an occupational therapist, and health sciences doctoral student with a background in translation (undergraduate in Japanese translation, with English as the second language).

The participants’ textual interview contents were numerically anonymized to safeguard data anonymity. The research team then read all the interview texts line by line, employing the inductive thematic analysis method to discern recurring content themes. Thematic analysis was chosen because it is suitable for questions related to people’s experiences, opinions, or perceptions and is a common method for identifying, reporting, and interpreting patterns in qualitative data (17). The research team individually reviewed each participant’s interview transcripts multiple times until no new information emerged, indicating data saturation. Previous studies have indicated that saturation can be achieved with as few as 9 to 17 interviews (18). After triangulating the data, the analysis team discussed and resolved discrepancies, identifying two primary data-driven themes: “changes in lifestyle habits,” and “changes in interpersonal relationships.” Under the theme of “changes in lifestyle habits,” several sub-themes were identified including “handwashing,” “mask-wearing,” “physical distancing,” “healthy regular diet,” “changes in sleep habits.” Certain themes were subsequently excluded due to insufficient reporting by participants or a weak evidential basis. Thereafter, all the interview texts were imported into NVivo QSR version 14. This software facilitated swift and effective manual coding and categorization in accordance with coding established by the research team.

### 2.5 Ethics statement

Ethical approval was granted by the Epidemiology Research Ethics Review Committee of Hiroshima University, and participants were informed of their right to withdraw at any stage. Prior to commencement, all the participants provided their written and verbal consent.

## 3 Results

This study conducted semi-structured interviews and inductive thematic analysis using NVivo software, to explore lifestyle changes among 20 international students at Hiroshima University, Japan, during the post-pandemic era. The analysis uncovered significant shifts in lifestyle habits and interpersonal relationships in the aftermath of the pandemic, which are discussed in detail.

### 3.1 Sample characteristics

Between April and June 2023, 20 international students were recruited from Hiroshima University. The cohort comprised 8 male and 12 female students, with an average age of 31.5 years. Detailed characteristics of the participants are presented in Table 1.

**Table 1:** Participants’ characteristics.

Gender	Nationality	N or Mean (SD)
Male	Bangladesh	5
	China	3
Female	Bangladesh	3
	China	9
Age (years)		31.5 (6.45)

### 3.2 Changes in lifestyle post-COVID-19

**Figure 1:**
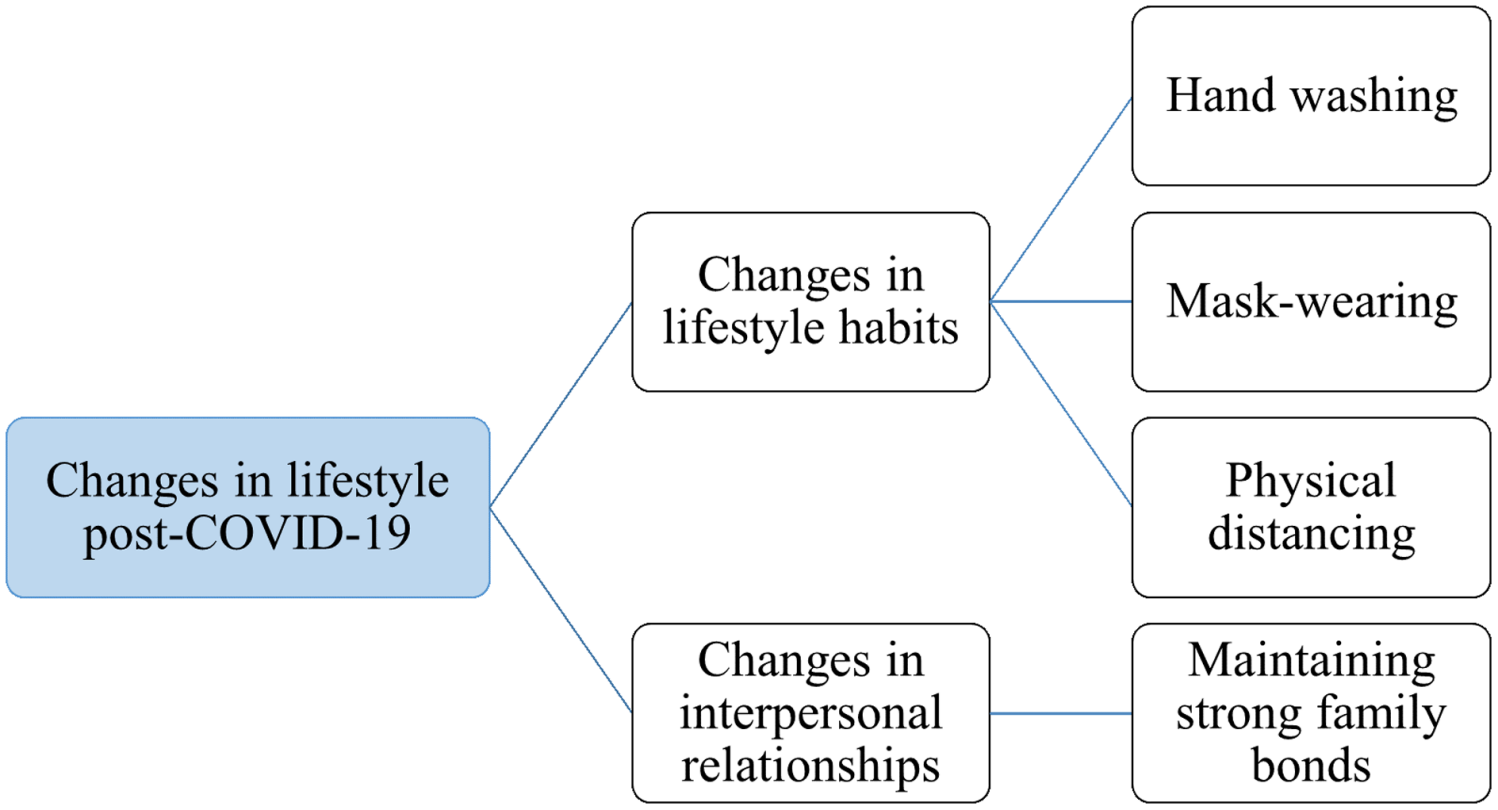
Changes in lifestyle post-COVID-19.

Based on the interview content, the research team found that the lifestyles of international students in the post-pandemic era had changed under the influence of epidemic prevention policies implemented by the Japanese government and schools. The research team identified two data-driven themes from the interview content: changes in lifestyle habits and changes in interpersonal relationships. Mentions by participants relating to “habits” were coded under the theme of “changes in lifestyle habits.” References to topics such as “placing greater emphasis on family ties” and “desiring closer intimacy with family” were coded under the theme of “changes in interpersonal relationships.” Figure 1 depicts the lifestyle changes observed in international students during the post-pandemic era.

#### 3.2.1 Changes in lifestyle habits

During the interviews, all participants mentioned adopting preventive health measures during COVID-19 to avoid infection, influenced by the epidemic prevention policies implemented by the Japanese government and schools. Post-pandemic, 17 (85%) participants indicated that their lifestyles had changed compared to pre-pandemic times, with a heightened focus on personal health. Some of the preventive health behaviors adopted during the pandemic had also become habitual. In contrast, the remaining 3 participants (15%) reported that their lifestyles had remained unchanged after the pandemic, and they did not maintain any health behaviors from that period. Table 2 lists the preventive health behaviors recommended by the Japanese government and schools during COVID-19, as reported by the respondents, while Table 3 details the continued adoption of these behaviors post-COVID-19.

**Table 2:** Epidemic prevention and health behaviors during the COVID-19 pandemic.

Prevention and health behaviors	Number
Obtaining epidemic-related information	19
Mask-wearing	17
Attending online classes	15
Hand washing	13
Home isolation	13
Physical distancing	6
Temperature measurements	4
Vaccination	1

**Table 3:** Epidemic prevention and health behaviors retained after the COVID-19 pandemic.

Prevention and health behaviors	Number
Hand washing	10
Mask-wearing	12
Physical distancing	4

Through comparing the preventive health behaviors of international students during and after COVID-19, it was found that changes in lifestyle habits primarily centered around three health measures: handwashing, mask-wearing, and physical distancing.

##### 3.2.1.1 Hand washing

Hand washing can effectively prevent COVID-19. The Japanese government and schools promoted frequent hand washing during the pandemic. Consequently, 13 participants adopted this preventive behavior during COVID-19. Of the 17 participants who reported changes in lifestyle habits post-pandemic, 10 continued to practice regular hand washing.

*“After the epidemic, I developed the habit of washing hands frequently.”* (Participant 17, female, China).

*“After the epidemic, I pay special attention to this aspect (healthy behavior). Washing hands and disinfecting have now become a habit.”* (Participant 19, female, China).

##### 3.2.1.2 Mask-wearing

Masks are effective in reducing the spread of respiratory viruses. Both the government and schools advocated mask-wearing as a preventive measure. During the pandemic, 17 participants adhered to wearing masks, and 12 continued this practice even after the end of COVID-19.

“*Before COVID-19, we did not wear masks although we had some air pollution in Dacca city, but after COVID-19, we mostly wear masks consciously.*” (Participant 7, male, Bangladesh).

*“Just like I feel about wearing glasses, I also feel about wearing a mask, which has now become a part of my attire.”* (Participant 2, female, Bangladesh).

##### 3.2.1.3 Physical distancing

Physical distancing is an important measure advocated by both the Japanese government and schools to control COVID-19. While 6 participants reported following physical distancing behavior during the pandemic, 4 reported that they retained this habit, even after the end of COVID-19.

“*After the epidemic, I pay more attention to hygiene. Maybe I did not have the concept of disinfection and keeping distance from others before.*” (Participant 13, female, China).

*“If I get sick, I might need to take a break from my education, which is why I always try to maintain my personal health, like trying to maintain distance from unknown persons…. So; that is the difference. Before COVID-19, I think I was not following this practice.”* (Participant 6, male, Bangladesh).

##### 3.2.1.4 Epidemic prevention and health behaviors that were not retained

During the pandemic, participants adopted various preventive health measures recommended by the government and schools, such as obtaining epidemic-related information, attending online classes, practicing home isolation, temperature measurements, and receiving vaccinations. However, these measures were not consistently continued post-pandemic.

Although most participants ceased seeking epidemic-related information after the pandemic, it is important to highlight that 19 participants actively sought such information during the pandemic. With the exception of one participant who avoided epidemic-related information due to anxiety, the majority utilized diverse channels for information acquisition, including the internet (*n* = 19), television (*n* = 6), newspapers (*n* = 4), and lectures (*n* = 1). The internet emerged as the primary source of information, with two participants specifically mentioning official websites like the World Health Organization. Seventeen participants reported using social media platforms, such as Twitter (X), TikTok, and Facebook for obtaining information. Additionally, vaccination was the least mentioned health preventive behavior.

### 3.3 Changes in interpersonal relationships

#### 3.3.1 Maintaining strong family bonds

During the interviews, researchers uncovered a theme pertaining to interpersonal relationships. Many international students encountered challenges in returning home due to the Japanese government’s epidemic prevention policies during the pandemic. Five participants expressed their intention to prioritize family and interpersonal relationships post-pandemic, emphasizing the elevated significance of familial bonds. The maintenance of strong family bonds emerged as their foremost priority in future planning.

*“Previously, I had thought about living abroad in the future, but now I feel it might be better to stay closer to my family. For example, during a pandemic, there might be sudden travel restrictions, and I might not be able to return home even if a family member passes away. Customs will not make exceptions for this. I just feel I want to be closer to my family.”* (Participant 18, female, China).

*“I was considering pursuing a Ph.D. in Europe or America, but owing to this pandemic, I now feel that if I am too far away from home and something happens, it would be very difficult to come back, especially considering that my parents are getting older.”* (Participant 16, female, China).

## 4 Discussion

This study examined lifestyle changes among international students in Japan during the post-pandemic period from April to June 2023, influenced by epidemic prevention policies implemented by both, schools and the government. It revealed shifts in both lifestyle habits and interpersonal relationships among the participants.

It found that the majority of international students persisted in maintaining epidemic prevention and health behaviors developed during the pandemic. Specifically, they continued to adhere to three key behaviors: handwashing, mask-wearing, and physical distancing even after the pandemic’s conclusion. These findings align with previous research on the formation of habitual preventive behaviors. Throughout the pandemic, individuals were encouraged to adopt protective measures. Repeated engagement in these behaviors in similar situations often leads to habit formation (19). Although handwashing, mask-wearing, and physical distancing were all personal hygiene behaviors during the pandemic, most studies on the impact of the epidemic on individual health behaviors only mention changes in hand hygiene post-pandemic (20–22). However, among international students in Japan, mask-wearing and physical distancing were also mentioned. This may be related to the influence of Japanese society.

During the pandemic, some individuals in certain countries rejected the notion of wearing masks, and even displayed negative attitudes toward mask-wearing (23). Consequently, the adoption of mask-wearing as a habitual practice post-pandemic is unlikely. A study conducted in Bangladesh also revealed widespread resistance to wearing masks, with the majority expressing reluctance to utilize them (24). However, Japan has a custom of wearing masks, making mask-wearing a common practice in the country (25). Influenced by this societal context, international students in Japan also adhere to the practice of wearing masks. Notably, in this study, mask-wearing emerged as the most entrenched habit among the three health behaviors. This may be attributed to handwashing being a pre-existing habitual behavior for many individuals. Conversely, mask-wearing, has become increasingly habitual over time (26).

Furthermore, a study regarding individual protective behaviors after vaccination campaigns across four countries found that personal protective measures significantly relaxed post-vaccination, particularly in social activities (27). Chinese university students were also found to exhibit decreased personal protective behaviors after receiving the COVID-19 vaccine (28). However, a study in Japan found that individuals who had completed vaccination did not decrease their personal protective behaviors (29). In contrast to their compatriots, international students in Japan have adopted personal protective behaviors similar to those of Japanese nationals. Even after the epidemic ended, they continued to take preventive measures, and made it a habit. Habit formation has played a pivotal role in individuals’ behavioral responses to preventive measures during COVID-19 (30). Education in personal hygiene and public awareness in Japanese elementary schools has contributed to Japanese people developing good hygiene habits (31). Public awareness is defined as the public’s understanding and comprehension of the risks of infectious disease. Increasing public awareness is crucial for controlling the spread of infectious diseases (32, 33). Compared to other countries, Japan has consistently implemented a national school health policy over many years (34). In Japan, the emphasis on public awareness begins in elementary school, and extends through junior high school (35). Consequently, Japanese individuals are equipped to make informed decisions and act when confronted with public emergencies. During COVID-19, Japanese citizens demonstrated a greater inclination than those in other countries to adhere to the infection prevention policies advocated by their government. Certain nations implemented public health measures through legislation to govern citizens’ preventive behaviors. In contrast, the Japanese government advocated the voluntary adoption of preventive measures among its citizens, relying on self-discipline to mitigate transmission of the virus. This could be attributed to public awareness levels in certain countries not being notably high (36). In Bangladesh, there are no educational programs or subjects at any level, including primary schools, focusing on public awareness (37). Therefore, increasing public awareness among international students in Japanese universities by offering public health courses is essential for ensuring their health and preparing them to effectively cope with potential future public health events.

Moreover, investigating health and preventive measures that were implemented during the pandemic, but subsequently discontinued without becoming habitual, can also provide insights into safeguarding the health and well-being of international students. During COVID-19, only one international student mentioned that vaccination is a preventive measure recommended by the Japanese government to prevent infection. However, after the pandemic, none of the international students retained the habit of receiving regular vaccinations as a preventive measure against infectious diseases. During COVID-19, the Japanese government offered free vaccination services to residents in Japan. Japan’s vaccination law encourages regular preventive vaccinations among its residents, and some local governments also provide subsidies for vaccination (38). A previous investigation into COVID-19 vaccination rates in Japan underscores, that the vaccination rate among foreign residents is lower than that among Japanese nationals (39). This may be because international students encounter numerous challenges when receiving medical services. A study has shown that migrants in Japan faced language barriers while scheduling appointments, completing questionnaires, and receiving vaccinations (40). The Japanese government primarily communicates information about vaccine administration and health check-ups to residents in Japan through postal mail, which is predominantly in Japanese. Although Japan started deploying interpreters in certain cities since September 2021 (41), interpreter availability at vaccine centers across Japan is not widespread. Therefore, addressing the language barriers encountered by international students when accessing government-provided medical services is essential for preserving their health and well-being. Another web survey targeting Vietnamese immigrants in Japan revealed the lack of knowledge about medical facilities and services available in Japan (42). Despite the Japanese government disseminating medical services-related information through multilingual websites, many international students are unfamiliar with the available channels for accessing this information. Instead, they tend to rely more on social media platforms. The majority of international students utilized social media platforms to seek information on the pandemic and health during the outbreak, with only a minority resorting to official websites. Post-pandemic, none of the international students maintained the practice of consistently seeking health-related information. However, unofficial websites may contain inaccurate information, and such misinformation can have significant adverse consequences for epidemic prevention efforts (43). Therefore, ensuring that international students receive timely and accurate medical services information is crucial for enhancing their health and well-being. Policymakers should address language barriers and information gaps that impede international students’ access to healthcare by providing multilingual health information and services. This approach will help ensure that these students have equitable access to vaccinations and other healthcare services.

Research examining post-COVID-19 lifestyle changes among adult populations typically emphasizes psychological health and health behaviors. However, contrary to earlier findings (44, 45), this study revealed shifts in interpersonal relationships between international students and their families following the pandemic. This change may be linked to family resilience, which refers to the family’s collective capacity as a functional system to endure and recover from adversity, aiding in navigating difficult periods and maintaining stability, thus fostering positive outcomes for all its members (46–48).

Many international students were unable to return to their home countries during the pandemic due to epidemic prevention measures, leading them to reassess their priorities. A heightened focus on maintaining family bonds emerged as a primary consideration in their future plans and career aspirations. Given the changes in international students’ post-epidemic plans, it is essential for institutions to provide career guidance and support tailored to the current context. This will facilitate their successful employment or further academic pursuits.

### 4.1 Limitations

Despite this study’s strengths, it has some limitations. The nationalities of the participants were all Chinese and Bangladeshi, and the results may not reflect the true situation of all international students, limiting the generalizability. The retrospective nature of the interview questions is another limitation, as it may be associated with recall bias. Furthermore, the assessment of international students’ lifestyle changes was limited to the period between April and June 2023. Therefore, subsequent research is imperative to ascertain whether the results of this study can be applied to international students from other countries and the observed changes persisted beyond this timeframe.

## 5 Conclusion

This study’s results indicate that, influenced by epidemic prevention policies implemented by schools and the government, there have been changes in the lifestyle habits and interpersonal relationships of international students in Japan in the post-pandemic era. Following the pandemic, the majority of international students have continued to habitually maintain certain epidemic prevention and health behaviors, such as mask-wearing, handwashing, and physical distancing, that they had adopted during the pandemic. However, some of these epidemic prevention and health behaviors have not persisted as habits post-pandemic. Furthermore, there is an increased emphasis on maintaining family bonds in the post-pandemic period. These findings offer insights into improving the health and well-being of international students. Educational institutions should implement public health courses aimed at enhancing international students’ public awareness, particularly regarding infectious disease risks. This knowledge is essential for safeguarding their health and equipping them to navigate potential public health crises effectively. Additionally, providing career and academic guidance that is responsive to contemporary needs represents a crucial step toward improving the overall health and well-being of international students. Furthermore, policymakers must consider language barriers and challenges in information dissemination when developing national public health policies. To address these issues, it is imperative to offer multilingual health information and services, ensuring that international students can access the health care services they need without impediments. This study’s findings are expected to provide essential references and foundations for developing targeted and effective health promotion measures aimed at enhancing the well-being of international students in the future.

## Data Availability

The raw data supporting the conclusions of this article will be made available by the authors, without undue reservation.
