# Supplementary material for: Enhancing the health and well-being of international students: insights from changes in their lifestyle post-COVID-19
Source: Front Public Health. 2024 Dec 17;12:1470378. doi: 10.3389/fpubh.2024.1470378 (PMC11685117; doi:10.3389/fpubh.2024.1470378)
Supplement: Supplementary file 1 [file Table_1.DOCX]

| Consolidated criteria for reporting qualitative studies (COREQ): 32-item checklist | | | | | | | | | |
| --- | --- | --- | --- | --- | --- | --- | --- | --- | --- |
| No Item |  |  |  |  |  |  |  |  |  |
| Domain 1: Research team and reflexivity | | | | | Guide questions/description | | | | |
| Personal Characteristics | | | | | | | | | |
| 1. Interviewer/facilitator | | | | | The first author conducted the interview (page 3, lines 35-37). | | | | |
| 2. Credentials | | | | | The first author is a PhD student in health sciences, and the rest of the research team includes two PhDs in medical fields and, one MD in health sciences fields. | | | | |
| 3. Occupation | | | | | The research team consisted of a doctoral supervisor with extensive experience in qualitative research and qualifications in psychiatry, university lecturer specializing in social welfare studies, university assistant with qualifications as an occupational therapist, and healthcare doctoral student with a translation background (undergraduate in Japanese translation, with English as the second language) (page 4, lines 26-30). | | | | |
| 4. Gender | | | | | The research team, composed of two males and two females (page 4, line 25). | | | | |
| 5. Experience and training | | | | | The first author had previously undertaken courses related to qualitative research, and this study was conducted under the guidance of a doctoral supervisor, possessing extensive experience in qualitative research and qualifications in psychiatry, for ensuring its credibility and validity (page 3, line 40-page 4, line 1). | | | | |
| Relationship with participants | | | | | | | | | |
| 6. Relationship established | | | | | There was no relationship established prior to study commencement. Participants were recruited using a snowball sampling method (page 3, lines 29-31). | | | | |
| 7. Participant knowledge of the interviewer | | | | | Before starting the interview, we provided participants with a brief introduction to the research purpose, significance, methods, and other related content (page 3, lines 38-40). | | | | |
| 8. Interviewer characteristics | | | | | The first author conducted the interview. The first author is a healthcare doctoral international student at Hiroshima University with a translation background (undergraduate in Japanese translation, second language is English). With a background in translation studies and fluency in three languages (Chinese, Japanese, and English), the first author encountered no significant language barriers during the interviews (page 3, lines 24-25; page 4, lines 4-5; page 4, lines 28-30). | | | | |
| Domain 2: study design | | | | | | | | | |
| Theoretical framework | | | | | | | | | |
| 9. Methodological orientation and Theory | | | | | Using induction and thematic analysis methods to determine the content theme. Thematic analysis was chosen because it is suitable for questions related to people's experiences, opinions, or perceptions and is a common method for identifying, reporting, and interpreting patterns in qualitative data (page 4, line 31-page 5, line 2). | | | | |
| Participant selection | | | | | | | | | |
| 10. Sampling | | | | | A snowball sampling method was employed with the expectation that participants who had accepted the invitation could refer additional eligible participants (page 3, lines 29-31). | | | | |
| 11. Method of approach | | | | | Since the first author is an international student at Hiroshima University, the research invitation letter was sent to the Hiroshima University international student chat group with the assistance of a Bangladeshi student who knows the primary author and a Chinese student who is also acquainted with the first author. Interested participants in the study were encouraged to contact the first author either by phone or email, using the contact information provided in the invitation letter (page 3, lines 24-29). | | | | |
| 12. Sample size | | | | | According to a previous study, sample sizes ranging from 6–8 participants are optimal for ensuring sample homogeneity, whereas sample sizes ranging from 12–20 persons may be optimal for ensuring heterogeneity. This study focused on a group of international students in Japan and did not wish to limit the examinations to a specific demographic characteristic (e.g., specific marital status, gender, or age), and could benefit from the richness of heterogeneous data. Therefore, 20 international students at Hiroshima University were recruited (Chinese students, n = 12 participants; Bangladeshi students n = 8 participants) (page 3, lines 19-24). | | | | |
| 13. Non-participation | | | | | None of the participants refused to participate or dropped out (page 3, lines 33-34). | | | | |
| Setting | | | | | | | | | |
| 14. Setting of data collection | | | | | Nineteen participants took part in face-to-face semi-structured individual interviews conducted at Hiroshima University. One participant gave birth during the study period and requested to conduct the interview over the phone from home (page 3, lines 33-35). | | | | |
| 15. Presence of non-participants | | | | | To ensure participant privacy, only the participants and researchers (no non-participants) were present during the interviews (page 3, lines 36-37). | | | | |
| 16. Description of sample | | | | | All participants are international students in Japan. 20 international students were recruited from Hiroshima University. The participants comprised 8 male and 12 female students, with an average age of 31.5 years. The nationalities of the participants in this study are Chinese and Bangladeshi (page 3, lines 21-22; page 5, lines 14-15; page 10, lines 2-3). | | | | |
| Data collection | | | | | | | | | |
| 17. Interview guide | | | | | The research team convened a meeting to discuss and develop a questionnaire. The interviews were conducted using this questionnaire, which consisted of six questions. The questionnaire enabled the researcher to ensure more systematic interview procedures for participants and to explore new areas of dialogue concurrently. One participant underwent a pilot test of the questionnaire, and after incorporating participant's feedback, the questionnaire was modified. (page 3, lines 13-15; page 4, lines 9-16) | | | | |
| 18. Repeat interviews | | | | | One participant was invited for a second interview (page 3, lines 14-15). | | | | |
| 19. Audio/visual recording | | | | | All interviews, conducted by the first author were audio-recorded with the participants' consent (page 3, line 38). | | | | |
| 20. Field notes | | | | | The first author took field notes during these interviews (page 3, lines 35-36). | | | | |
| 21. Duration | | | | | The interviews lasted from 0.5 hours to 1 hour, and the length varied according to personal circumstances and experiences (page 4, lines 17-18). | | | | |
| 22. Data saturation | | | | | The research team individually reviewed each participant's interview transcripts multiple times until no new information emerged, indicating data saturation. Additionally, previous studies have indicated that saturation can be achieved with as few as 9 to 17 interviews (page 4, lines 36-38). | | | | |
| 23. Transcripts returned | | | | | Not implemented. | | | | |
| Domain 3: analysis and findings | | | | | | | | | |
| Data analysis | | | | | | | | | |
| 24. Number of data coders | | | | | After triangulating the data, the analysis team discussed and resolved discrepancies, identifying two primary data-driven themes: "changes in lifestyle habits," and "changes in interpersonal relationships." Under the theme of "changes in lifestyle habits," several sub-themes were identified: "handwashing," "mask-wearing," "physical distancing," "healthy regular diet," "changes in sleep habits," and so on. Subsequently, certain themes were excluded owing to insufficient reporting by participants or weak evidential basis (page 4, lines 39-44). | | | | |
| 25. Description of the coding tree | | | | | Based on the interview content, the research team found that the lifestyles of international students in the post-pandemic era had changed under the influence of epidemic prevention policies implemented by the Japanese government and schools. From the interview content, the research team identified two data-driven themes: changes in lifestyle habits and changes in interpersonal relationships. In the interviews, mentions by participants relating to "habits" were coded under the theme of "changes in lifestyle habits." References to topics such as "placing greater emphasis on family ties" and "desiring closer intimacy with family" were coded under the theme of "changes in interpersonal relationships." Figure 1 depicts the lifestyle changes observed in international students during the post-pandemic era (page 5, lines 18-25). | | | | |
| 26. Derivation of themes | | | | | All the theme is data driven (page 5, lines 20-21). | | | | |
| 27. Software | | | | | All the interview texts were imported into NVivo QSR version 14. This software facilitated swift and effective manual coding and categorization in accordance with coding established by the research team (page 4, line 44-page 5, line 2). | | | | |
| 28. Participant checking | | | | | Not implemented. | | | | |
| Reporting | | | | | | | | | |
| 29. Quotations presented | | | | | Implemented. | | | | |
| 30. Data and findings consistent | | | | | Confirmed. | | | | |
| 31. Clarity of major themes | | | | | Confirmed. | | | | |
| 32. Clarity of minor themes | | | | | Confirmed. | | | | |
